# Molecular Mechanisms of Colorectal Liver Metastases

**DOI:** 10.3390/cells12121657

**Published:** 2023-06-18

**Authors:** Diamantis I. Tsilimigras, Ioannis Ntanasis-Stathopoulos, Timothy M. Pawlik

**Affiliations:** 1 Department of Surgery, Division of Surgical Oncology, The Ohio State University Wexner Medical Center and James Comprehensive Cancer Center, 395 W. 12th Ave., Columbus, OH 43210, USA; 2 Department of Clinical Therapeutics, School of Medicine, Alexandra General Hospital, National and Kapodistrian University of Athens, 11527 Athens, Greece; johnntanasis@gmail.com

**Keywords:** CRLM, cells, tumor microenvironment

## Abstract

The liver is the most frequently target for metastasis among patients with colorectal cancer mainly because of the portal vein circulation that directly connects the colon and rectum with the liver. The liver tumor microenvironment consists of different cell types each with unique characteristics and functions that modulate the antigen recognition and immune system activation. Primary tumors from other sites “prime” the liver prior to the seeding of cancer cells, creating a pre-metastatic niche. Following invasion into the liver, four different phases are key to the development of liver metastases: a microvascular phase in which cancer cells infiltrate and become trapped in sinusoidal vessels; an extravascular, pre-angiogenic phase; an angiogenic phase that supplies oxygen and nutrients to cancer cells; and a growth phase in which metastatic cells multiply and enlarge to form detectable tumors. Exosomes carry proteins, lipids, as well as genetic information that can create a pre-metastatic niche in distant sites, including the liver. The complexity of angiogenic mechanisms and the exploitation of the vasculature in situ by cancer cells have limited the efficacy of currently available anti-angiogenic therapies. Delineating the molecular mechanisms implicated in colorectal liver metastases is crucial to understand and predict tumor progression; the development of distant metastases; and resistance to chemotherapy, immunotherapy, and targeted treatment.

## 1. Introduction

Colorectal cancer (CRC) is one of the three most common cancers worldwide. According to the American Cancer Society, approximately 153,000 individuals will be diagnosed with CRC and 52,500 will die from the disease in the United States in 2023 [1]. Despite advances in treatment of patients with CRC, up to 50% will develop colorectal liver metastases (CRLM) during the course of their disease [2,3,4]. The liver is the most frequent location for metastasis among patients with CRC mainly because of the portal vein system that drains the colon and rectum through the liver. In turn, CRLM remains the main cause of death among patients with CRC [4]. 

Resection and chemotherapy are the mainstay of treatment for patients with CRLM. Nevertheless, only 10–20% of CRLM cases are amenable to resection at the time of diagnosis [5]. Among patients with resectable disease, 5-year post-resection survival ranges between 20–50% and is dependent on a wide range of factors, including age of patient, presence of comorbidities, as well as tumor size and location [4]. Patients who are not deemed surgical candidates have an even worse prognosis [6]. Despite the advancement and the optimization of chemotherapeutic schemes, CRLM patients treated with fluorouracil and platinum-based chemotherapy will eventually develop toxicity and side effects as well as resistance, resulting in suboptimal outcomes [7]. As such, it is imperative that more effective targeted therapies are developed that will improve survival of patients with CRLM in the long-term. Understanding the complex molecular mechanisms implicated in the process of CRLM development and chemoresistance may contribute toward this goal. The current review provides an overview of cellular interactions and the main mechanisms implicated in the development of liver metastases.

## 2. Molecular Mechanisms of Liver Metastases

**Figure 1:**
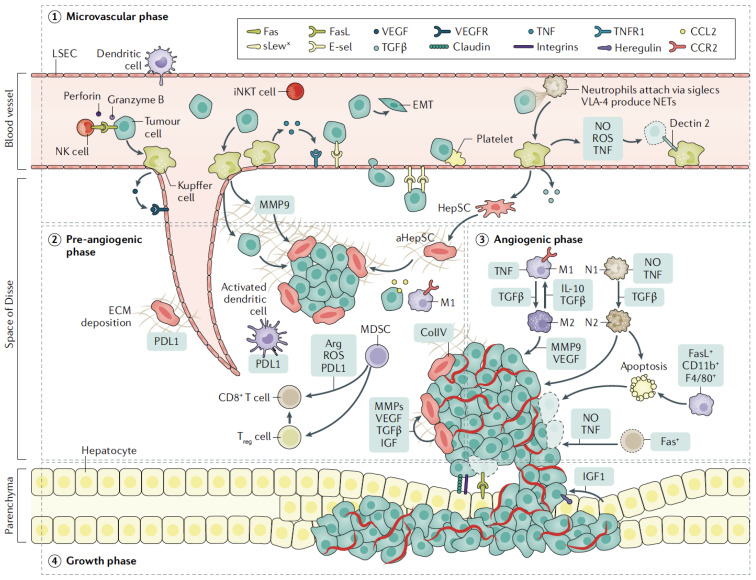
Schematic representation of phases involved in liver invasion by tumor cells. Upon entering the liver within sinusoidal vessels (microvascular phase), tumor cells encounter resident Kuppfer cells, LSECs, and NK cells, which lead to early cell death or extravasation of cells into the space of Disse. HepSCs are activated by factors secreted by Kuppfer cells, including TGFβ and secreted ECM, that provide the means for endothelial cell migration (pre-angiogenic phase). Neo-vascularization begins mainly mediated by VEGF produced by tumor cells or activated Kuppfer cells (angiogenic phase). Immune cells are recruited with either immune-suppressing or tumor-promoting properties, while expansion of CD8+ T cells is impaired by expression of PDL1. In turn, tumor cells attach to hepatocytes that release growth factors, facilitating tumor expansion (growth phase). Abbreviations: TNF: tumor necrosis factor; CCL2: C-C motif ligand 2; NO: nitric oxide; ROS: reactive oxygen species; EMT: epithelial–mesenchymal transition; iNKT, invariant natural killer; T cell; MMP: matrix metalloproteinase; IGF: insulin-like growth factor; collIV: collagen type IV; Fas Reproduced with permission from ref. [4].

CRC cells seed the liver through the portal venous circulation. In contrast, other GI tract cancer cells may seed the liver through the systemic circulation via the hepatic artery [8]. Upon entry of CRC cells to the liver, there are four basic steps implicated in the process of liver metastases. These include: A. a microvascular phase where cancer cells infiltrate and become trapped in sinusoidal vessels, B. an extravascular, pre-angiogenic phase, C. an angiogenic phase that supplies oxygen and nutrients to cancer cells, and D. a growth phase where metastatic cells multiply and enlarge to form detectable tumors. Specific interactions between cancer cells and other liver cells, both recruited and resident, occur at each phase. (Figure 1) [9,10].

## 3. Cellular Approach to Liver Metastasis

**Figure 2:**
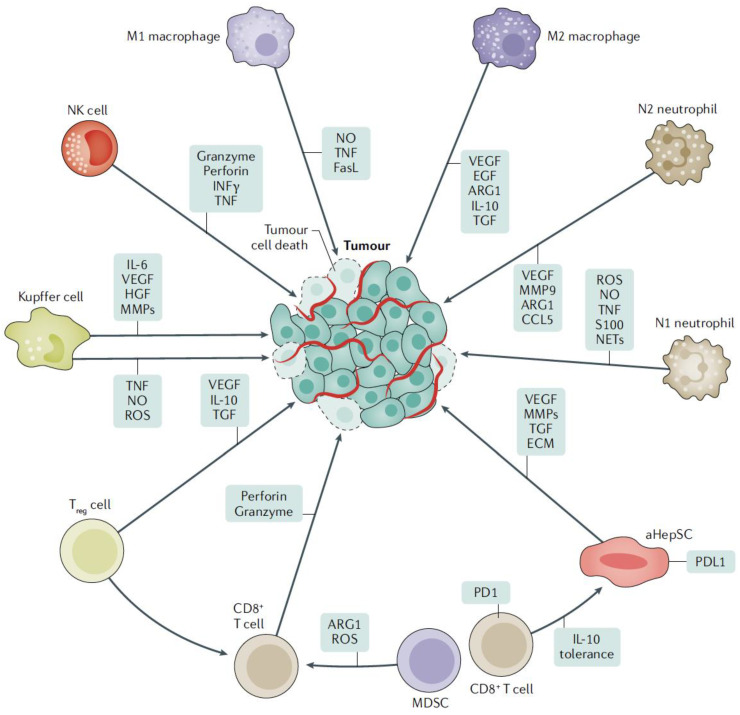
Cellular interactions between cancer and immune cells. Certain cells may inhibit or promote cancer cell growth, depending on their polarization (e.g., M1 and M2 macrophages). Through major histocompatibility complex interactions, multiple cells in the liver present antigens, leading to upregulation of PLD1 expression and, in turn, exhaustion of the T cell compartment as well as immune tolerance. MDSC deplete and Tregs suppress CD8+ T cells, thus an immunosuppressive microenvironment is formed. Reproduced with permission from [4].

Upon reaching the liver, metastatic cancer cells interact with multiple highly specialized resident cells that form a unique microenvironment [11,12,13]. This microenvironment consists of parenchymal hepatocytes, liver sinusoidal endothelial cells (LSECs), Kupffer cells (KC), hepatic stellate cells (HepSC), dendritic cells (DC), as well as resident natural killer (NK) cells [8,14]. Each cell type has distinct characteristics that enable them to perform specific metabolic functions and to modulate the immune response to antigen stimuli that enter the liver via the portal circulation from the gut (Figure 2) [11,12,13,14].

### 3.1. Hepatocytes

Roughly 60% of the liver consists of hepatocytes that are responsible for its synthetic function [15]. These specialized cells contain numerous mitochondria and abundant endoplasmic reticulum, which facilitate the production of coagulation factors, albumin, as well as other serum proteins required for liver metabolism and detoxification of various chemicals. After liver injury, hepatocytes can proliferate rapidly to enable the liver to regenerate, although the exact mechanism of liver regeneration still remains under investigation [16]. In particular, it is not clear whether the process is governed by stem or progenitor cells, or replication of residing hepatocytes [16]. Among other mechanisms, hepatocytes have been shown to express integrins to trap tumor cells via osteopontin or form desmosomes with cancer cells, thereby allowing these cells to accumulate in the liver [17].

### 3.2. LSECs

The sinusoidal vessels are lined with LSECs. The distinctive fenestrations allow circulating lymphocytes to interact with the underlying hepatocytes by exposing the hepatocytes and hepSCs in the Disse space to blood components [12,18].

### 3.3. Kupffer Cells

About 10% of all liver cells are Kupffer cells, a specific kind of liver cell that is found in the sinusoids of the liver [19]. These cells are crucial for the metabolism of cholesterol, the eradication of infections, and the activation of local immunity [19]. Progenitors of Kupffer cells are formed from yolk sac precursors during the early stages of embryonic development and go to the liver. These cells are continuously replaced to preserve homeostasis after they have established themselves in the liver. Scavenger receptors, Toll-like receptors, and nucleotide-binding oligomerization domain-like receptors (NLRs), among others, are expressed on the cell surfaces of mature Kupffer cells and enable for the detection and eradication of infections and apoptotic cells [20]. When Kupffer cells are activated, they produce cytokines and chemokines, including TNFb and IL-1, that signal other immune cells to dangerous infections [20].

### 3.4. NK Cells

Under normal circumstances, the liver contains a substantial number of NK cells that play an important role in resisting infections and eliminating cancer cells [21,22]. These NK cells act as a surveillance system to detect potential threats to the liver that lack self-cell markers, allowing the cells to identify and destroy pathogens and cancer cells. This process is particularly important because some cancer cells evade adaptive immunity by downregulating antigen-presenting molecules such as MHC-I [21]. In addition, the liver also contains a distinct NK cell population known as thymus-derived invariant NK T cells (iNKT) that are characterized by their expression of CD4 and CD8 molecules [23,24].

### 3.5. HepSCs

The HepSCs comprise approximately 15% of the non-parenchymal liver cells and are responsible for coordinating the hepatic response to injury and inflammation. These cells are typically inactive and reside in the space of Disse yet become activated when the liver sustains an injury. When activated, HepSCs transform into a type of myofibroblast (known as SMA+ aHepSC) and produce an extracellular matrix (ECM) consisting of collagens I and IV, which promotes fibrosis [25,26]. In addition, HepSCs release chemokines that attract other immune cells and also form the liver immune microenvironment [27,28].

### 3.6. Dendritic Cells

Several subtypes of dendritic cells have been identified in the liver, including myeloid dendritic cells (mDCs), which express CD11b, CD11c, and CD1c; and plasmacytoid dendritic cells (pDCs), which express CD123 and are CD11c- and BDCA-2 (CD303)+ in humans [18]. Although their activities are not yet completely known, mice also exhibit CD11c+CD8+ and CD11c+NK1.1+ populations of lymphoid-related dendritic cells [18,29,30].

### 3.7. Monocytes and Neutrophils

Monocytes and neutrophils can be recruited to the liver in response to liver injury, inflammatory stimuli, or in response to invasion by metastatic cancer cells [31,32]. For example, CCR2highLy6Chigh monocytes are commonly present in a variety of liver diseases yet are also present in the context of liver metastases [33,34,35]. In contrast, neutrophils can be recruited to sites of inflammation by expressing a number of cell surface markers, including CD44, siglec-9 and -10, and VLA-4, which connect to counter receptors on LSECs to facilitate migration [36,37].

Hepatocytes, LSECs, Kupffer cells, HepSCs, and dendritic cells may act as antigen-presenting cells to recruited T cells. Subsequently, immune tolerance can develop via upregulation of PD-L1 and interaction of PD-1 with T cells. This process may lead to T cell exhaustion and the secretion of immunosuppressive molecules such as IL-10 or TGF [20,29,38]. Such an immune-tolerant condition can promote a pro-metastatic niche and could be one of the reasons for the suboptimal response to immunotherapy among patients with liver metastases [39,40,41].

## 4. Formation of a Pre-Metastatic Niche in the Liver

Liver metastases develop in two main phases: A. the pre-metastatic niche that is formulated by secreted factors stemming from the primary tumor, and B. the post-tumor invasion niche that is formed following the entry of tumor cells in the liver [42]. The first stage is characterized by the deposition of circulating cancer cells in the sinusoids and capillaries, later followed by transit and proliferation in the liver parenchyma. This process disrupts the normal function of liver cells and leads to the creation of a microenvironment that favors tumor growth [42]. For example, hematopoietic bone marrow progenitor cells tend to accumulate in the liver prior to tumor invasion [43,44]. Those cells express extracellular matrix molecules such as MMP-9 that facilitate metastatic seeding and contribute to the formation of a “premetastatic niche”. Tumor cells may also promote an intravascular, premetastatic niche by activating endothelial cells, leading to the expression of adhesion factors such as E-selectin and VCAM1 [45,46]. Activation of endothelial cells results in the release of cytokines such as TNF-α, IL-1-β via IL-18 signaling [47]. The interactions between tumor cells and the endothelium may impair the integrity of the endothelial junctions, facilitating the migration of cancer cells into the liver parenchyma [48]. Exosomes (i.e., cancer-derived microvesicles) containing nucleic acids and proteins have been thought to play a key role in the formation of a premetastatic niche [49,50,51]. In addition, cancer cells tend to attach to platelets, which protect them from shear stress and NK cells. Of note, disrupting tumor cell–platelet interactions by inhibiting COX1 and its downstream effector TXA2 with aspirin has been noted to inhibit liver metastases in experimental models [48].

## 5. Interactions of Cancer Cells with Liver Microenvironment

### 5.1. Tumor Cells First Encounter LSEC and Kupffer Cells

LSECs and resident Kupffer cells, which are found in the sinusoids, are the first cells that tumor cells make contact with when they penetrate the liver. At that point, cancer cells may be eliminated quickly due to mechanical stress and shear forces within sinusoids. In addition, Kupffer cells can eliminate tumor cells via phagocytosis via their unique receptors, such as Dectin-2; therefore, they might have a protective role against metastatic seeding [52]. Kupffer cells and LSECs also express TNFα, nitric oxide (NO), and reactive oxygen species (ROS), which can induce cancer cell death. Hepatic NK cells also induce cancer cell apoptosis by the release of perforin/granzyme or by activating the Fas/FasL pathway [9,10,53]. Following cancer cell death, several cytokines (i.e., IL-1, IL-6, IL-8, IL-12, IL-18) and chemokines such as CCL5 are released that lead to the mobilization and activation of additional immune cells, further contributing to the host’s defense against cancer [54,55]. Nevertheless, the resultant local inflammatory response can induce the expression of LSEC adhesion receptors that mediate cancer cell adhesion directly or indirectly, thereby allowing cancer cells to transmigrate into the space of Disse. Furthermore, cancer cells may be protected from apoptotic signals deriving from Kupffer cells by activating the signaling pathways, including the p38, MAPK, and ERK pathways. Importantly, LSECs may potentiate the metastatic potential through facilitating epithelial-mesenchymal transition, which allows tumor cells to escape from innate immune cells through increased migration and invasion [56,57]. Kuppfer cells are predominantly tumoricidal in early phases, yet the cells can promote metastasis in the latter phase through the secretion of growth factors that enhance cancer cell proliferation (i.e., HGF) and angiogenesis (i.e., VEGF) [55,58]. Kupffer cells can also recruit other cells that promote metastasis, including macrophages and MDSCs, that in turn lead to the creation of an immune-tolerant microenvironment [59]. On the other hand, LSECs have an immunosuppressive effect which disables the immune-mediated anti-tumor response to metastatic cancer in the liver [29,60].

### 5.2. Tumor Cell Interaction with Neutrophils

Neutrophils are one of the first myeloid cells to interact with cancer cells prior to their extravasation. These cells may have dual functions, promoting or inhibiting metastasis [32,61]. Neutrophils can release NO, ROS, TNF, TNF-related apoptosis-inducing ligands (TRAIL), and cytolytic defensins to promote cancer cell death. Neutrophils can also recruit CD8+ cytotoxic T cells and macrophages [62]. Neutrophils can also contribute to cancer cell retention within the vessels by forming neutrophil extracellular traps (NETs) that can trap circulating cancer cells, leading to accelerated growth at those sites and, thus, increased metastatic potential [63]. Neutrophils can have pro- or anti-tumor effects, depending on differentiation or polarization states (i.e., N1 and N2), which are regulated by the tumor microenvironment and specifically TGFβ and IGF-1 [32,61,64,65,66]. In liver metastases, neutrophils express FGF2, which can play a role in angiogenesis and tumor progression [67]. In other organs, neutrophils can exhibit an anti-tumor immune response via the production of iNOS and by inhibiting IFN production by CD8 T cells, as seen in murine breast cancer liver metastases [68,69].

### 5.3. Tumor Cell Interaction with Recruited Macrophages

Liver metastases attract host myeloid cells, which have a crucial role in inducing metastatic potential. This recruitment of monocytes and macrophages to liver metastatic sites is triggered by pro-inflammatory states that involve TNF, CCL2, CCL9, and CCL15 [33,34,70]. Blocking the CCL2-CCR2 signaling axis can reduce macrophage infiltration, ultimately resulting in less or no metastatic expansion [34,35]. Macrophages can shift functionally from M1 to M2, with M1 macrophages being pro-inflammatory and exerting tumoricidal effects, while M2 macrophages typically promote tumor growth by producing growth factors, including VEGF, FGF2, and TGFβ. Furthermore, macrophages can release MMPs that contribute to increased invasion and metastatic expansion while inhibiting the host immune response and inducing regulatory T cells (Treg), which promote cancer cell survival [71]. Eventually, the monocytes attracted to liver metastatic sites develop into CD11b+F4/80+ macrophages that stimulate angiogenesis by secreting cytokines, including VEGF and TGF [72,73]. Mechanisms of M1 and M2 phenotype macrophages are quite complex; both M1 macrophages (TNF producing pro-inflammatory) and M2 macrophages (VEGF-producing) can directly or indirectly contribute to liver metastasis. As such, strategies to eliminate macrophages in vivo to inhibit cancer growth are still under investigation [9,55,74].

### 5.4. MDSCs Have a Pro-Metastatic Potential

MDSCs comprise a diverse population of precursor cells, including granulocytic (G-MDSC) and monocytic (Mo-MDSC) subtypes. In response to chemokines secreted by Kuppfer cells, LSECs, or HepSCs (i.e., CXCL1, CXCL2), MDSCs migrate from the bone marrow to the liver [28,75,76]. The factors responsible for preventing MDSC differentiation into mature neutrophils or macrophages in the tumor microenvironment, thereby maintaining their immunosuppressive function, are not yet completely understood. Nevertheless, studies suggest that VEGF, GM-CSF, G-CSF, and M-CSF may be involved in promoting myelopoiesis and inhibiting myeloid cell maturation [77,78]. However, immature MDSCs have an immunosuppressive function by inhibiting NK cell activity and blocking the adaptive T cell response through the production of arginase, which consumes the L-arginine in the microenvironment, an important amino acid required for T cell survival and function [78,79,80,81,82,83]. In addition, MDSCs produce ROS that cause T cell apoptosis and recruit Treg cells through the production of CCL5. Enhanced STAT3 pathway activation mediated by estrogen receptor alpha in response to cytokines like IL-6 may control the formation of MDSC in the tumor microenvironment. MDSC accumulation has been associated with JAK2 and SRC activation in human and mouse bone marrow myeloid precursors and murine colorectal cancer liver metastasis models [84,85]. CD11b, Ly6G, and Ly6C are commonly used cell surface markers for identifying MDSCs, although these markers are also present in fully differentiated neutrophils and macrophages [77]. The exact role of MDSCs in promoting metastatic expansion requires further investigation.

### 5.5. Tumor Cell Interaction with HepSCs

HepSCs can be activated in response to tissue injury following exposure to products released by apoptotic cells, as well as via the release of TGFβ, TNF, and granulin from macrophages. Subsequently, this may initiate a process similar to liver regeneration/repair [86,87]. HepSCs produce a type of extracellular matrix (ECM) that is rich in type I and IV collagen. HepSCs also release growth factors such as TGFβ and proangiogenic factors, including VEGF and angiopoietin-1, as well as chemokines and cytokines comprised of CCL2, CCL5, and CCL21 [28,88,89], which attract inflammatory or immune cells. In addition, enzymes such as MMP-2, MMP-9 and MMP-13 are produced to facilitate ECM turnover [90,91,92]. Angiogenesis, mediated primarily by VEGF and angiopoietin-1, is further enhanced by the release of ECM as well as MMPs that facilitate endothelial cell migration and cancer cell invasion [9,74,86,93,94]. HepSCs can also present antigens to T cells, which can trigger an adaptive immune response against cancer cells. Nevertheless, antigen presentation by HepSCs preferentially leads to immune tolerance via the PD-L1/PD-1 pathway and the induction of Treg cells [95,96]. Furthermore, HepSCs may induce the conversion of monocytes to MDSCs in a CD-44-dependent fashion, which further contributes to the immunosuppressive tumor microenvironment [97].

### 5.6. Tumor Cell Interaction with Hepatocytes

Although the exact role of hepatocytes in the development and progression of metastatic cancer to the liver remains unclear, recent research suggests that hepatocytes may contribute to the development of a pro-metastatic environment. Tumor cells can adhere to hepatocytes by interacting with desmosomes [98]. Furthermore, integrins such as α6, αv, and β1 may mediate attachment of tumor cells to the ECM of hepatocytes [99]. Hepatocytes also bind to osteopontin via corresponding receptors CD44 and integrin αv, while claudins interact with hepatocytes through trans-homotypic claudin-2 interactions [100,101]. Interactions of tumor cells to hepatocyte-derived ECM may upregulate gene expression that promotes survival, motility, and proliferation of cancer cells, such as heparin-binding epidermal growth factor (HB-EGF) and the colon stem cell marker leucine-rich repeat-containing G protein-coupled receptor 5 (LGR5) [102]. In turn, cancer cells can activate Fas signaling to induce apoptosis of hepatocytes, thus facilitating metastatic expansion [103]. Additionally, hepatocytes produce growth factors such as insulin-like growth factor-1 (IGF-1), hepatocyte growth factor-like protein (HGFL), and heregulin, which can enhance tumor growth, motility, and invasion [104,105], while also triggering angiogenesis by inducing basic fibroblast growth factor (bFGF) production [9,106].

## 6. Role of Exosomes in Establishing Pre-Metastatic Niche in the Liver

Accumulating evidence suggests that exosomes—membrane vesicles of endocytic origin ranging in size from 30 to 150 nm—play a key role in intercellular communication between cancer cells and their microenvironment. These microvesicles can be released from both cancerous and non-cancerous cells and act as carriers of functional contents such as proteins, lipids, and RNA molecules (mRNAs, lncRNAs, miRNAs) and circulate DNA from donor to recipient cells [107,108]. The exchange of contents between microvesicles and cancer cells, which occurs through fusion, alters the functions of the recipient cells [107,108]. Tumor-derived microvesicles can create pre-metastatic niches in distant sites, facilitating metastasis. For example, in a murine model of pancreatic adenocarcinoma (PDAC), secreted exosomes were shown to activate Kuppfer cells, leading to increased TGF-β production, as well as HepSCs, leading to the deposition of the extracellular matrix to the liver via macrophage-inhibition factor [109]. For example, high exosomal MIF levels have been associated with higher rates of liver metastases among individuals with early stage PDAC [109]. Similarly, among patients with CRLM, microvesicles contain miR-21, which upon integration into resident macrophages such as Kupffer cells results in IL-6 synthesis and reverts the microenvironment to a proinflammatory state [110]. Of note, increased miR-21 levels in exosomes derived from individuals with CRC have been associated with a higher potential of developing liver metastases [110]. As such, plasma exosomes could be useful in the risk stratification of cancer patients relative to their risk of developing metastasis.

## 7. Role of Angiogenesis in Developing of Liver Metastases

Angiogenesis is essential for providing oxygen and nutrients necessary for the growth of primary tumor and metastases. Although targeting angiogenesis has been a promising approach to cancer therapy, it has only had limited success to date. Targeted therapies against VEGF and its receptors have been FDA-approved for the treatment of patients with CRLM. However, some studies have noted that the liver may become more susceptible to metastasis post-treatment due to changes in liver sinusoid morphology and increased vascular permeability that occur following anti-VEGF therapy [111,112]. Certain molecules, such as FGF-2 secreted by neutrophils recruited to the liver, can promote liver growth of metastasis and vascular remodeling [67]. Vascular co-option—whereby metastatic cells use pre-existing tissue blood vessels rather than neo-angiogenesis—also promotes tumor cell survival [113]. In fact, the microscopic evaluation of liver metastasis specimens revealed that the invasive edges of these tumors were associated with pre-existing blood vessels, which may contribute to the clinical refractoriness of liver metastases to anti-angiogenic therapy [113]. Indeed, anti-VEGF therapy seems to have minimal effect on preventing liver metastases in malignancies that rely on vascular co-option for nutrient supply, particularly those tumors with a replacement growth pattern [113]. In turn, the complexity of angiogenic mechanisms, and the preferrable use of pre-existing vessels by cancer cells, have limited the efficacy of currently available anti-angiogenic therapies.

## 8. Conclusions

The liver metastatic cascade is a complex, multi-step biologic process. The tumor microenvironment is pivotal in tumor progression and metastatic invasion. Mitigating pro-tumorigenic while enhancing anti-tumorigenic functions of the tumor microenvironment can maximize the efficacy of novel targeted therapies. Delineating the molecular mechanisms implicated in liver metastases is key to understanding and predicting tumor progression, the development of distant metastases, and resistance to chemotherapy. Further research is needed to identify factors within the tumor microenvironment that could potentially represent suitable targets for novel therapeutic approaches.

## Data Availability

Not applicable.
